# First record of Podocarpoid fossil wood in South China

**DOI:** 10.1038/srep32294

**Published:** 2016-08-30

**Authors:** Long Li, Jian-Hua Jin, Cheng Quan, Alexei A. Oskolski

**Affiliations:** 1 State Key Laboratory of Biocontrol, Guangdong Provincial Key Laboratory of Plant Resources, and School of Life Sciences, Sun Yat-sen University, Guangzhou 510275, China; 2 Research Center of Paleontology & Stratigraphy, Jilin University, Changchun 130026, China; 3 Department of Botany and Plant Biotechnology, University of Johannesburg, Auckland Park 2006, Johannesburg, South Africa; 4 Komarov Botanical Institute of the Russian Academy of Sciences, Prof. Popov str. 2, St. Petersburg 197376, Russia

## Abstract

A new species of fossil conifer wood, *Podocarpoxylon donghuaiense* sp. nov., is described from the late Eocene of Nadu Formation in Baise Basin of the Guangxi Province, South China. This fossil wood is characterized by distinct growth rings, circular to oval tracheids in cross section, 1–2-seriate opposite pits on radial tracheid walls, uniseriate (rarely biseriate) rays, smooth end walls of ray parenchyma cells, and the absence of resin ducts, suggesting its affinity to Podocarpaceae. The new species is distinctive from other Cenozoic woods ascribed to this family by the combination of distinctive growth rings, the absence of axial parenchyma, the occurrence of bordered pits on tangential tracheid walls, and the occurrence of 3–4 cuppressoid or taxodioid pits on cross-fields. This represents the first record of podocarpoid fossil wood in South China and provides fossil evidence for the early dispersal and diversification of Podocarpaceae in eastern Asia as well as for mild temperate seasonal climate in this region during the late Eocene.

As the second largest family within the conifers, the modern Podocarpaceae largely comprises evergreen trees and shrubs belonging to 194 species within 19 genera^1^. This family is mainly distributed in tropical and subtropical regions from central to South America, Africa (include Madagascar), Indochina through Malesia to Australia and Oceania^1^,^2^, extending as far north as China and Japan as well as to Mexico and the Caribbean^3^,^4^,^5^. Podocarpaceae are most abundant in montane tropical rainforests, but occasionally occur in lowland forests^6^. In comparison with extensive investigations on extant phylogenetics and geography of the family, its early evolution and migration in deep time are still poorly known, mainly due to the lack of megafossils, especially of Cenozoic age.

Fossil records of podocarps from the Mesozoic were well documented in both the Northern and Southern hemispheres. Molecular and fossil evidence suggests that the podocarps originated during Triassic-Jurassic time in Gondwana and apparently spread to the Northern Hemisphere during the Jurassic^7^. Although the family appears to be of ancient origin, molecular dating analysis suggests that the majority of extant genera have arisen relatively recently in the Upper Cretaceous to Cenozoic^8^.

Although modern Podocarpaceae are widespread mostly in the Southern Hemisphere, there are numerous reports of Cenozoic megafossils and palynomorphs attributable to this family from the Northern Hemisphere. *Podocarpus* shoots were found in Eocene deposits of Tennessee^9^, whereas pollen seemingly belonging to this genus is known from the Miocene of Oregon and Idaho^10^ and late Eocene of Colorado^11^. In Europe, leaves of *Prumnopitys* have been reported from the Eocene of England^12^, while leaf fossils assigned to *Podocarpus* have been found in Eocene to Oligocene deposits of Ukraine^13^,^14^ and of the southeastern regions of European Russia^15^. A phylloclade of *Phyllocladus* has also been described by Krasnov^13^ from Eocene deposits of the Kharkov region (Ukraine). The assignment of these fossils to *Podocarpus* and *Phyllocladus* need special examination, however. In particular, several records of *Podocarpus*-like leaves from Eocene to Miocene localities of central and eastern Europe ascribed to the species *Podocarpus eocenica* Ung. have been reconsidered by Ferguson *et al.*^16^ as members of *Amentotaxus*, a genus of the Taxaceae. In addition, podocarpoid fossil wood has been reported from the Oligocene-Miocene of southern Ural^17^. Podocarpaceae palynomorphs occur widely in Europe from the Paleocene to the Miocene^18^,^19^,^20^, whereas the records of *Podocarpus* pollen from the Pliocene of Iceland^21^ and Pleistocene of Belgium^21^,^22^ possibly result from redeposition from older beds^19^.

In Asia, several fossil woods attributed to *Podocarpoxylon* are known from the early Tertiary to Pliocene of India^23^. *Podocarpus* leaves have been reported from the Oligocene of Assam^24^, while *Nageia* leaves have also been described from Eocene deposits of Hainan Island and Guangdong Province, South China^25^,^26^. Podocarpaceae fossil wood of Cenozoic age has not been recorded in China before but several apparent podocarpaceous woods, including *Podocarpus (Nageia*) *nagi* Pilger^27^ have been reported from Lower Cretaceous deposits^28^,^29^. As for microfossils, Paleocene palynological records of Podocarpaceae locally occur in India, but they are very poorly represented in Southeast Asia where the pollen of *Podocarpus* sensu lato appears in the late Eocene^7^. In northern Kazakhstan, Podocarpaceae pollen grains have also been reported from the Eocene, but they disappear there in the late Oligocene^30^.

Here we describe a new fossil wood of *Podocarpoxylon* from the late Eocene Nadu Formation of Baise Basin, Guangxi Province of South China, and review the phytogeographic and ecological implications of our finding. This is the first occurrence of fossil Cenozoic Podocarpaceae wood from South China.

## Results

### Systematics

Order: Araucariales Gorozh.

Family: Podocarpaceae Endl.

Genus: *Podocarpoxylon* Gothan, 1908.

Species: *Podocarpoxylon donghuaiense* Li, Jin, Quan et Oskolski, sp. nov.

### Etymology

The specific name “Donghuai” is the locality name where the fossils were collected.

### Holotype

DHW006.

### Paratypes

DHW001 to DHW005, DHW007 to DHW0013.

### Repository

Fossil wood samples and microscopic slides are deposited in the Museum of Biology, Sun Yat-sen University, Guangzhou, China.

### Type locality and horizon

**Figure 1:**
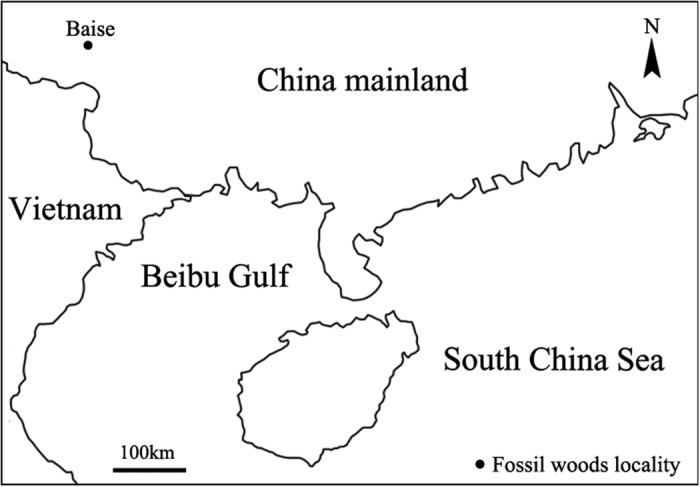
Location of the study area. Location of Baise Basin, Guangxi Province, South China. (drawn by L.L., using Adobe Photoshop CS5).

Nadu Fm., late Eocene. Specimens were collected in Donghuai coal-mine. Baise City, Guangxi Province (Fig. 1).

### Diagnosis

Growth rings with prominent latewood, transition from earlywood to latewood gradual; circular pits on radial tracheid walls uniseriate, sometimes biseriate opposite, circular pits rarely occur in tangential walls of tracheids; cross-field pits cupressoid and taxodioid, circular to oval, 1–4 pits (mean 2) in opposite 1–2 rows per cross-field; axial parenchyma absent; rays predominately uniseriate, rarely partially biseriate; ray cells with smooth horizontal and tangential walls; ray tracheids absent; resin ducts absent.

### Description

**Figure 2:**
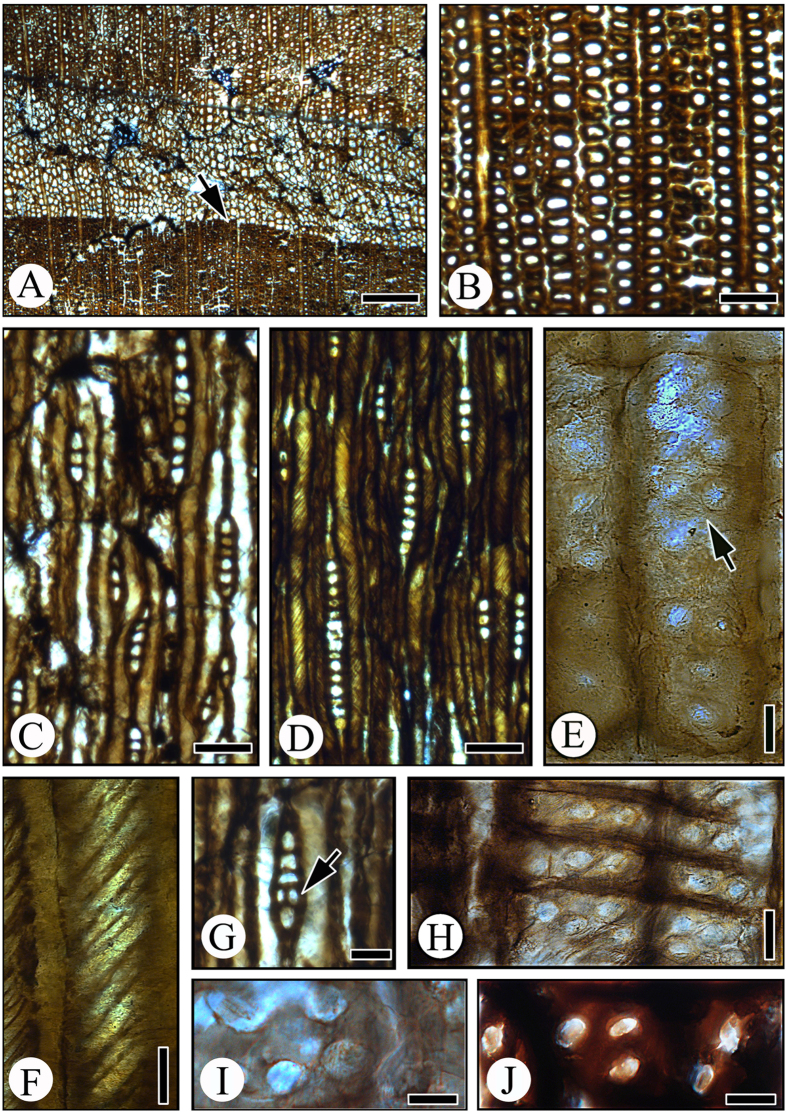
Wood structure of *Podocarpoxylon donghuaiense* sp. nov. (DHW006). (**A**) Transverse section showing distinct growth rings (arrow). Scale bar = 400 μm. (**B**) Transverse section of early wood showing circular to oval tracheids. Scale bar = 100 μm. (**C**) Tangential section showing the predominately uniseriate rays. Scale bar = 100 μm. (**D**) Tangential section of late wood showing spiral thickenings in tracheids (arrow). Scale bar = 100 μm. (**E**) Radial section showing 1–2 seriate opposite pits in radial tracheid walls (arrow). Scale bar = 20 μm. (**F**) Radial section showing spiral and branched thickenings on tracheid walls. Scale bar = 20 μm. (**G**) Tangential section showing partially 2-seriate rays (arrow). Scale bar = 50 μm. (**H**) Radial section showing cupressoid and taxodioid pits on cross-fields. Scale bar = 20 μm. (**I**) Radial section showing cupressoid pits on cross-fields. Scale bar = 10 μm. (**J**) Radial section showing taxodioidpits on cross-fields. Scale bar = 10 μm.

Growth rings distinct, 5.2–7.9 mm wide, with prominent latewood, transition from earlywood to latewood gradual (Fig. 2A). Earlywood tracheids thin-walled, circular to oval in cross-sectional outline, 23–40 μm (mean 32 μm) in tangential diameter and 23–56 μm (mean 37 μm) in radial size (Fig. 2B). Latewood tracheids moderately thick-walled, circular to oblong in cross-sectional outline, 7–23 μm (mean 13 μm) in tangential diameter and 11–21 μm (mean 15 μm) in radial size.

Pits in radial tracheid walls uniseriate, occsionally also biseriate opposite (Fig. 2E). Pits bordered, circular (11–17 μm in diameter with the average of 15.7 μm) to oval (14–20 μm in size) in outline. Crassulae absent. Bordered pits of 8.2–15.0 μm in diameter rarely occur also on the tangential walls of latewood tracheids. Well-developed tightly spaced spiral and branched thickenings present on radial and tangential walls of latewood tracheids (Fig. 2D,F), with angles to the tracheid axes ranging from 40° to 60°.

Axial parenchyma absent.

Rays 42–331 μm (mean 100 μm) in height, predominately 1-seriate (Fig. 2C,D) and rarely partially 2-seriate (Fig. 2G), completely composed of parenchyma cells, 1–16 cells (mean 5 cells). Ray cells oval or elliptical in tangential section, both vertical and horizontal walls of ray parenchyma cells smooth, indentures absent. Cross-field pits possibly cupressoid or taxodioid type, circular to oval of 6–12 μm in size, with 1–4 pits (mean 2) per cross-field (Fig. 2H–J). Crystals not found. Resin ducts absent.

## Discussion

### Comparison with modern materials

Within the conifer families, the fossil wood from Donghuai may not be placed into Pinaceae, Cupressaceae or Cephalotaxaceae, as it has no resin ducts, axial parenchyma and ray tracheids. This late Eocene wood is also distinctive both from Araucariaceae by the absence of two or more seriate alternate intertracheary pitting and by the lacking crowded araucarioid cross-field pits, and from Sciadopityaceae by the absence of window-like cross-field pits. Therefore, it remains for us to consider the relationships of this fossil wood to Taxaceae or Podocarpaceae^31^,^32^,^33^,^34^.

The presence of spiral and branched thickenings on the walls of latewood tracheids in combination with smooth horizontal and tangential walls of ray cells suggests the fossil wood from Donghuai may be considered as a member of Taxaceae^31^,^32^,^33^,^34^. After careful examination of the thickenings on tracheid walls in this sample we suggest, however, that these structures are not of the same nature as the spiral tertiary thickenings that occur in all modern genera of this family, with the exception of *Austrotaxus* R.H. Compton^31^,^34^. Extant Taxaceae show finer spiral thickenings, more widely spaced and tilted at lower angles (usually not exceeding 45°) in respect to the tracheid axes than the thickenings observed in the late Eocene wood from Donghuai. Such features of the fossil wood as tracheids bearing spiral thickenings confined only to the latewood and their absence in the earlywood has also not been reported in any modern Taxaceae^31^,^32^,^33^,^34^. Therefore, the spiral and branched structures occurring in tracheids of the Donghuai wood are more likely artifacts (probably, effects of wood compression) than true tertiary thickenings. The occurrences of similar artifacts in fossil woods seemingly belonging to Taxaceae have been surveyed by Afonin & Philippe^35^. In fossil woods assigned to Podocarpaceae these structures have been reported by Chudajberdyev^17^ in tracheids of *Podocarpoxylon uralense* Chudajb.

As far the thickenings on tracheid walls are recognized as artifacts rather than diagnostic traits, the suite of other characters (smooth horizontal and tangential walls of ray cells without indentures, cupressoid and taxodioid pits on cross fields) indicates that the fossil wood reported here has greatest affinity to the Podocarpaceae^31^,^32^,^33^,^34^. This family, however, shows considerable diversity of wood structure that does not allow distinction between its genera using wood anatomical traits. The late Eocene wood from Donghuai is different from most modern Podocarpaceae in growth rings with conspicuous latewood, lacking axial parenchyma and the occurrence of 1–4 pits on the cross-field. However, the presence of prominent latewood portion has been reported in growth rings of *Podocarpus acutifolius* Kirk, *Podocarpus macrophyllus* (Thunb.) D. Don., *Lagarostrobus (Dacrydium*) *franklinii* (Hook. f.) Quinn, *Halocarpus (Dacrydium*) *bidwillii* (Hook. f. ex T. Kirk) Quinn, *Phyllocladus glaucus* Carr. and *P. trichomanoides* D. Don^32^,^34^,^36^,^37^,^38^. Axial parenchyma is also lacking in *Dacrydium elatum* (Roxb.) Wall. ex. Hook., *D. colensoi* Hook., *D. biforme* (Hook.) Pilg., *D. kirkii* F. Muell. ex Parl., *D. intermedium* Kirk, *D. taxifolium* Banks & Sol. ex D. Don, *Halocarpus bidwillii Lepidothamnus intermedius* (Kirk) Quinn., *Manoao colensoi* (Hook.) Molloy., *Microcachrys tetragona* (Hook.) Hook., *Phyllocladus alpinus* Hook.f.*, P. glaucus, P. trichomanoides*, *Podocarpus elongatus* (Aiton) L’Hér. ex Pers., *Prumnopitys harmsiana* (Pilg.) de Laub., and *P. taxifolia* (Sol. ex D.Don) de Laub^31^,^32^,^33^,^36^,^37^,^39^,^40^. Although cross-fields with 1–2 pits are the most common condition in Podocarpaceae, the occurrence of cross-fields with up to 4 cupressoid or taxodioid pits has been reported for *Dacrydium pierrei*, *D. intermedium*, *Podocarpus hallii*, *Microcachrys tetragona* and *Retrophyllum minor* (Carrière) C. N. Page^32^,^34^,^36^,^37^,^39^. Therefore, the fossil wood from Donghuai shows most resemblance to some species of *Prumnopytis* (especially *P. taxifolia*) as well as to some members of *Podocarpus*, *Dacrydium* and *Microcachrys*, but it cannot be convincingly placed in any extant genus of Podocarpaceae on the basis of its anatomical traits.

### Comparison with fossil materials

The fossil wood described here is characterized by an absence of axial parenchyma and by the cross-fields with cupressoid and/or taxodioid pits. Within fossil woods ascribed to Podocarpaceae, these traits are found in some species of the genus *Podocarpoxylon* Gothan^31^,^32^,^41^, as well as in the monospecific genus *Prumnopityoxylon* Franco & Brea that was recently described from the Upper Cenozoic of Argentina^42^. Moreover, this combination of wood traits has also been reported for *Phyllocladoxylon annulatus* Patton from the Oligocene of Australia^43^. The fossil sample from Donghuai exhibits similarities to Cenozoic species of *Podocarpoxylon* as well as with *Prumnopityoxylon gnaedingerae* Franco & Brea and *Phyllocladoxylon annulatus* Patton (Table 1).

Within seven species that have no axial parenchyma (*Prumnopityoxylon gnaedingerae* Franco & Brea, *Podocarpoxylon aparenchymatosum* Gothan, *P. dusenii* Kräusel, *P. latrobensis* Greenwood, *P. sahnii* (Ramanujam) Trivedi & Srivastava, *P. tiruvakkaraianum* (Ramanujam) Trivedi & Srivastava, and *Phyllocladoxylon annulatus* Patton), cross-fields with more than two pits occur only in *P. aparenchymatosum* and *P. gnaedingerae. Podocarpoxylon aparenchymatosum* from the Eocene deposits of Antarctica^44^,^45^ differs, however, from the Donghuai wood in possessing 3-seriate pitting on radial tracheid walls. Unlike *Prumnopityoxylon gnaedingerae*, the Donghuai wood sample shows distinct growth rings, and by higher rays height with the occasional occurrence of bi-seriate portions.

It follows from its unique character combinations that the late Eocene wood from Donghuai can be recognized as a new species named here as *Podocarpoxylon donghuaiense*. Although this fossil wood shows certain similarity to *Prumnopityoxylon*, Franco & Brea’s^42^ diagnosis of this genus lacks sufficient detail to separate it from *Podocarpoxylon*. Any of the wood traits, that have been considered by these authors as diagnostic for *Prumnopityoxylon* (i.e. “slightly distinct or indistinct growth rings; absence of axial parenchyma; uniseriate and homocellular rays; uniseriate or biseriate, opposite or sub-opposite, separate or contiguous tracheid pitting; taxodioid or cuppressoid cross-field pitting, with 1–5 bordered pits per field”)^42^, can be also found elsewhere in *Podocarpoxylon*, and their occurrences are consistent with Gothan’s^44^ diagnosis of this genus. For this reason, we ascribe the new species to *Podocarpoxylon* rather than to *Prumnopityoxylon*.

### Biogeographic implications

*Podocarpoxylon donghuaiense* sp. nov. is the first record of podocarpoid fossil wood in China. Coeval macrofossils assigned to Podocarpaceae have already been reported from the South China: well-preserved leaves of *Nageia* have recently been described from the Eocene Changchang Formation of Hainan Island, and the Eocene Youganwo and Huangniuling formations of Guangdong Province^25^,^26^. *Podocarpoxylon donghuaiense* is distinct, however, from the wood of extant *Nageia* species by the absence of axial parenchyma^32^,^33^,^34^. Thus it is very unlikely that the fossil wood from Guangxi belonged to the same plant taxon as the fossil leaves from Hainan and Guangdong, despite the geographical proximity and nearly the same age of these three findings.

Jin *et al.*’s^25^ and Liu *et al.*’s^26^, as well as our data confirm that the common occurrence of the Podocarpaceae species in the late Eocene vegetation of South China, that is consistent with the age of initial appearance of this family in Southeast Asia estimated by palynological records^7^. As the results of molecular dating suggest^8^, diversification of Podocarpaceae during Eocene gave rise to such modern genera distributed now in this region, as *Dacrydium*, *Podocarpus* and *Nageia*. Although *Podocarpoxylon donghuaiense* shares some wood traits with some extant species of *Podocarpus* and *Dacrydium*, this fossil wood can be convincingly ascribed to neither of them, probably because these taxa had not yet been emerged as distinct entities in the late Eocene.

Most modern species of Podocarpaceae have growth rings with indistinct to very narrow latewood, or growth rings lacking. Contrastingly, the growth rings of *Podocarpoxylon donghuaiense* show relatively high proportion of latewood with gradual transition from earlywood to latewood. These traits are indicative for plants with long growing period without rapid shift to seasonal dormancy that are encountered mainly in the middle latitudes of both hemispheres^46^,^47^,^48^. Within modern Podocarpaceae, this type of growth rings has been reported only in six species (*Podocarpus macrophyllus* ranged from Myanmar through mainland China and Taiwan to Japan, *Podocarpus acutifolius*, *Halocarpus bidwillii, Phyllocladus glaucus and P. trichomanoides* from New Zealand, and *Lagarostrobus franklinii* from Tasmania^49^) which are restricted to the temperate regions without dry season, with hot or warm summer (*Cfa and Cfb* climate types of Köppen’s classification)^50^. The occurrence of Podocarpaceae wood with prominent latewood in distinctive growth rings may therefore be an indicator that South China had mild temperate seasonal climate during the late Eocene.

## Conclusion

A new species of fossil conifer wood, *Podocarpoxylon donghuaiense* sp. nov., is described from the late Eocene of Baise Basin of Southern China. This is the first Cenozoic record of podocarpoid fossil wood in Southern China. The new species is distinguished from other Cenozoic woods ascribed to Podocarpaceae by the unique combination of distinctive growth rings, the absence of axial parenchyma, the lack of bordered pits on tangential tracheid walls, and the occurrence of 3–4 cuppressoid or taxodioid pits on cross-fields. The tightly spaced thickenings on latewood tracheid walls of the fossil wood are recognized as artifacts (probably, effects of wood compression) rather than true tertiary thickenings. Therefore, *Podocarpoxylon donghuaiense* sp. nov. provides the first robust physical evidence for the early steps of dispersal and diversification of Podocarpaceae in eastern Asia, as well as for warm and wet climate in this region, and the presence of clear growth rings also suggest this was a seasonal climate during the late Eocene.

## Methods

The fossil woods described here were collected from the Nadu Formation outcropping at Donghuai Coal-mine in the west part of the Baise Basin (transliterated also as the Bose Basin by some authors), Guangxi, South China (23° 52′ 14.84″N, 106° 34′ 49.27″E, Fig. 1). The geological age of the formation is believed to be late Eocene based on the well-studied Nadu Mammalian Fauna^51^.

The well-preserved wood specimens examined represent portions of a main trunk (DHW001-013). The holotype DHW006 is about 10 cm diameter by 15 cm in length. Thin-sections were prepared according to standard methods of cutting, grinding, and polishing using different grades of carborundum powder^52^. Wood slides were examined using a Carl Zeiss Axio Scope A 1 light microscope. Microphotographs were taken using a Cool Snap digital camera fitted with QCapture Pro 6.0 photographic software. Wood anatomical characters were measured and described according to the IAWA list of microscopic features for softwood identification^53^. The systematic position of the fossil wood was determined by consulting the key to morphogenera of fossil conifer woods^54^ and by carefully comparing with similar modern and fossil woods. Fossil wood generic nomenclature and circumscription followed the criteria of Philippe & Bamford^54^. Fossil wood specimens and thin sections are housed in the Museum of Biology, Sun Yat-sen University, Guangzhou, China.

## Additional Information

**How to cite this article**: Li, L. *et al.* First record of Podocarpoid fossil wood in South China. *Sci. Rep.* **6**, 32294; doi: 10.1038/srep32294 (2016).

## Figures and Tables

**Table 1:** Comparison of the fossil wood from Baise Basin with Cenozoic species of *Podocarpoxylon* and other selected taxa

Species	Age	Locality	Growth rings	Axial parenchyma	Pitting on radial walls, diameter of pits	Pitting on tangential walls	Ray width (cells)	Ray height (cells)	Number and diameter of pits on cross-field	Reference
Wood from Donghuai	Eocene	China	distinct	absent	1(2)-seriate 11–20 μm	+	1(2)	1–16	1–4 6–12 μm	
*Phyllocladoxylon annulatus* Patton	Oligocene	Australia	distinct	absent	1(2)-seriate 12–18 μm	+	1	1–7	1–2 8–13 μm	Patton^43^
*Podocarpoxylon aegyptiacum* Kräusel	Oligocene	Egypt	distinct	abundant	1-2-seriate	?	1	3-6	1-few	Kräusel^55^
*P. angustiporosum* E. Schönfeld	Eocene	Germany	distinct	sparse	1-2-seriate <18 μm	+	1	?	1–2(4) 9–12 μm	Schönfeld^56^
*P. aparenchymatosum* Gothan	Eocene	Antarctica	distinct	absent	1-3-seriate 11–19 μm	−	1	1–20	1–2(3)	Gothan^44^ Pujana *et al.*^45^
*P. articulatum* Süss & Velitzelos	Lower Miocene	Greece	distinct	sparse	1(2)-seriate 20–25 μm	?	1–2(3)	1–20 (100)	1–3 10 μm	Süss & Velitzelos^41^
*P. australe* Kräusel	Tertiary	Australia	indistinct to absent	sparse	1(2)-seriate 12–18 μm	+	1(2)	1–12	1–3 5–12 μm	Kräusel^57^; Patton^43^
*P. bruxellense* Stockmans	Eocene	Belgium	distinct	abundant	1(2)-seriate	?	1(2)	2–26	1	Stockmans^58^
*P. dacrydioides* Zalewska	Tertiary	Poland	indistinct	abundant	1–2(3)-seriate 13 μm	+	1(2)	1–31	1(2) 8 μm	Zalewska^59^
*P. dusenii* Kräusel	Tertiary	Argentina	distinct	absent	1-seriate	−	1(2)	1–20 (40)	1–2	Kräusel^60^
*P. graciliradiatum* Süss & Velitzelos	Lower Miocene	Greece	distinct	relatively abundant	1(2)-seriate 18–20 μm	?	1	1–30 (70)	1–3 10–12 μm	Süss & Velitzelos^41^
*P. jurii* Blokhina	Eocene-Oligocene	Russia (Kuril islands)	distinct	sparse	1(2)-seriate	?	1(2)	1–21	1–5	Blokhina^61^
*P.* aff. *javanicus* Merrill.	Pliocene	Georgia	distinct	sparse	1-seriate	?	1	1–12	1	Shilkina^62^
*P. kubarti* Rössler	Pliocene	Austria	indistinct	sparse	1-seriate	?	1(2)	< 23	1–4	Rössler^63^
*P. kurilense* Blokhina	Eocene-Oligocene	Russia (Kuril islands)	distinct	sparse	1-seriate	?	1	1–17	1–2	Blokhina^61^
*P. kutchensis* Lakhanpal, Guleria & Awasthi	Pliocene	India	indistinct	sparse	1(2)-seriate	?	1(2)	1–41	1–2	Lakhanpal, Guleria & Awasthi^64^
*P. latrobensis* Greenwood	Miocene	Australia	indistinct	scanty to absent	1-2-seriate 15 μm	−	1	2–18	1 (2)	Greenwood^65^
*P. mahabalei* (Agashe) Trivedi & Srivastava	Miocene-Pliocene	India	distinct	abundant	1-seriate	?	1	1–30	1	Trivedi & Srivastava^66^
*P. mazzonii* (Petriella) Müller-Stoll & Schultze-Motel	Paleocene	Argentina	indistinct	sparse	1-2-seriate	+	1(2)	1–38	1–2	Müller-Stoll & Schultze-Motel^67^
*P. minor* Patton	Oligocene	Australia	indistinct	sparse	1-seriate 8–13 μm	+	1	1–7	1–3 2.5–7.5 μm	Patton^43^
*P. sahnii* (Ramanujam) Trivedi & Srivastava	Miocene-Pliocene	India	distinct	absent	1-seriate	?	1(3)	1–20	1–2	Trivedi & Srivastava^66^
*P. schmidianum* Sahni	Tertiary	India	indistinct	sparse	1-2-seriate ca. 20 μm	?	1(2)	2–36 (100)	1–2	Sahni^68^
*P. speciosum* (Ramanujam) Trivedi & Srivastava	Miocene-Pliocene	India	distinct	abundant	1-2-seriate	?	1(2)	1–18	2–4	Trivedi & Srivastava^66^
*P. tiruvakkaraianum* (Ramanujam) Trivedi & Srivastava	Miocene-Pliocene	India	indistinct	absent	1-2-seriate	?	1	3–50	1	Trivedi & Srivastava^66^
*P. turoviense* Zalewska	Tertiary	Poland	distinct	abundant	1-2-seriate 15–17 μm	+	1(2)	1–45	1–2	Zalewska^59^
*P. uralense* Chudaib.	Oligocene_Miocene	Russia (south Ural)	distinct		1-seriate 13–14 μm	−	1	1–12	2–3 (5)	Chudajberdyev^17^
*P. vikramii* Bande & Prakash	Tertiary	India	indistinct	sparse	1(2)-seriate 15–20 μm	?	1	1–42	1–2 10–15 μm	Bande & Prakash^69^
*P. welkittii* Lemoigne & Beauchamp	Tertiary	Ethiopia	distinct	?	1(2) –seriate ca. 15 μm	?	1	1–18	1–3	Lemoigne & Beauchamp^70^
*P. yallournensis* Patton	Oligocene	Australia	indistinct	relatively abundant	1(2) –seriate 12–18 μm	+	1	1–6	1–3(5) 3–10 μm	Patton^43^
*Podocarpus falcatus* R. Br. ex Mirb.	Cenozoic	South Africa	indistinct	sparse	1(2) -seriate	?	1	3- >12	1	Adamson & Currin^71^
*Podocarpus* sp.	Pliocene	Columbia	indistinct to absent	abundant	1-seriate 8–17 μm	?	1	1–7	1 9–13 μm	Wijninga^72^
*Prumnopityoxylon gnaedingerae* Franco & Brea	Pliocene-Pleistocene	Argentina	indistinct	absent	1(2)-seriate 13–17 μm	+	1	2–8	1–5 3–9 μm	Franco & Brea^42^
